# Low-to-Moderate Arsenic Exposure and Urothelial Tract Cancers with a Long Latent Period of Follow-Up in an Arseniasis Area

**DOI:** 10.1007/s44197-023-00152-x

**Published:** 2023-09-19

**Authors:** Pei-Ju Liao, Chih-Hung Lee, Shu-Li Wang, Hung-Yi Chiou, Chien-Jen Chen, Chen-June Seak, I-Wen Wu, Kuang-Hung Hsu

**Affiliations:** 1 International Program of Health Informatics and Management, Chang Gung University, Taoyuan, Taiwan; 2 Master Degree Program in Health and Long-Term Care Industry, Chang Gung University, Taoyuan, Taiwan; 3 Division of Nephrology, Chang Gung Memorial Hospital, Keelung, Taiwan; 4 Department of Dermatology, Kaohsiung Chang Gung Memorial Hospital, Kaohsiung, Taiwan; 5 National Institute of Environmental Health Sciences, National Health Research Institutes, Miaoli, Taiwan; 6 Institute of Population Health Sciences, National Health Research Institutes, Zhunan, Miaoli County, Taiwan; 7 Master Program in Applied Epidemiology, College of Public Health, Taipei Medical University, Taipei, Taiwan; 8 Genomics Research Center, Academia Sinica, Taipei, Taiwan; 9 Department of Emergency Medicine, New Taipei Municipal Tucheng Hospital, New Taipei City, Taiwan; 10 Department of Emergency Medicine, Lin-Kou Medical Center, Chang Gung Memorial Hospital, Taoyuan, Taiwan; 11 College of Medicine, Chang Gung University, Taoyuan, Taiwan; 12 Laboratory for Epidemiology, Department of Health Care Management, and Healthy Aging Research Center, Chang Gung University, No. 259, Wen-Hwa 1St Road, Kwei-Shan, Taoyuan, 333 Taiwan; 13 Research Center for Food and Cosmetic Safety, College of Human Ecology, Chang Gung University of Science and Technology, Taoyuan, Taiwan; 14 Department of Safety, Health and Environmental Engineering, Ming Chi University of Technology, New Taipei City, Taiwan

**Keywords:** Long latency arsenic exposure, Bladder cancer, Upper urothelial tract cancers, Methylation capacity, Dose–response relationship

## Abstract

**Background:**

Arsenic exposure can cause adverse health effects. The effects of long-term low-to-moderate exposure and methylations remain unclear.

**Objective:**

This study aims to examine the association between low-to-moderate arsenic exposure and urothelial tract cancers while considering the effects of methylation capacity.

**Methods:**

In this study, 5,811 participants were recruited from an arseniasis area in Taiwan for inorganic arsenic metabolite analysis. This follow-up study was conducted between August 1995 and December 2017. We identified 85 urothelial tract cancers in these participants, including 49 bladder and 36 upper urothelial tract cancer cases. A Cox proportional hazards model was employed.

**Results:**

The analyses revealed a significant association between concentrations of inorganic arsenic in water > 100 ug/L and bladder cancer occurrence, with a hazard ratio (HR) of 4.88 (95% CI 1.35–17.61). A monotonic trend was observed between concentrations of inorganic arsenic in water (from 0 to > 100 ug/L) and the incidence of urothelial tract cancer, including bladder cancer (*p* < 0.05) and upper urothelial tract cancers (*p* < 0.05). Participants with a lower primary methylation index or higher secondary methylation index had a prominent effect.

**Conclusions:**

Rigorous regulations and active interventions should be considered for populations with susceptible characteristics.

## Introduction

The element arsenic is an environmental contaminant found in soil, air, food, and water. Chronic exposure to high levels of inorganic arsenic (iAs) is associated with numerous adverse effects, such as skin lesions, vascular diseases, cancers, reproductive toxicity, and neurological effects. According to the literature, bladder and kidney cancer mortality resulting from arsenic exposure has a long latency, with increased risks not manifesting until 40 years after exposure to arsenic [1]. The International Agency for Research on Cancer (IARC), based on compelling evidences, indicates that arsenic increases the risk of urinary bladder cancer. Studies have demonstrated that the association between exposure to iAs in drinking water and bladder cancer is detectable after exposure to iAs concentrations exceeding 100 μg/L. A prospective cohort study in northeastern Taiwan reported that the multivariate-adjusted relative risk of urinary tract cancer was statistically significant for residents who drank well water containing arsenic at levels > 100 μg/L [2, 3]. Results from different geographical areas have indicated a threshold for the association between iAs exposure at concentrations of 100 μg/L or higher and bladder cancer [4–6]. The risks related to exposure to arsenic below 10–100 μg/L remain unclear [7, 8]. In studies on lower arsenic exposure conducted in the United States, the association between populations consuming drinking water containing iAs at concentrations lower than 100 μg/L and bladder cancer incidence was found to be established [8–10]. Multiple epidemiological studies have supported that exposure to low concentrations of iAs may not increase the risk of bladder cancer [6, 11].

On the other hand, a study demonstrated an association between low-to-moderate levels of arsenic in drinking water and bladder cancer risk in New England [12]. In addition, a review article characterizing the risks in populations exposed to low concentrations of arsenic identified a potential association between bladder cancer risk and exposure to arsenic at concentrations of less than 100 μg/L in drinking water [13]. An ecological study demonstrated that a low concentration arsenic may be associated with lung and bladder cancers [14]. However, evidence in the form of individual data with dose–response relationship remains lacking.

Methylation is the central issue concerning inorganic arsenic carcinogenesis in humans. Studies have suggested that a higher ratio of methylarsonic acid (MMA) to dimethylarsinic acid (DMA) in urine is associated with an increased risk of developing bladder cancer [15, 16]. Studies have demonstrated that the risk of developing skin and bladder cancer increases with the percentage of methylated arsenic in urine [17, 18]. Furthermore, the overall risk of bladder (OR = 1.79; 95% CI 1.42–2.26, *n = *4 studies) and lung (OR = 2.44; 95% CI 1.57–3.80, *n = *2 studies) cancers increased significantly with the increase in %MMA without statistical heterogeneity [19]. In addition, a Taiwanese study demonstrated that subjects with a lower methylation capacity were more likely to develop a dose–response relationship between iAs exposure below the low-to-moderate range and lung cancer [20]. However, the effects of low-to-moderate arsenic exposure and its methylation on urothelial tract cancers occurrence are waiting for investigations. To close the gap of current knowledge, this study hypothesizes that the relationship between low-to-moderate iAs exposure and urothelial tract cancers may be moderated by iAs methylation capacity under a long latent period of observation.

## Methods

### Study Participants

This study was approved by the Institutional Review Board of Chang Gung Memorial Hospital in Taoyuan, Taiwan (100-2839C). On the basis of our previously established cohort in areas experiencing arseniasis, 5811 residents who were exposed to arsenic-contaminated well water at home and were eligible for a urinary iAs speciation analysis were defined as the study cohort. Residents of the arseniasis area in Northeast Taiwan were recruited for this study between 1992 and 1995. This prospective follow-up study was conducted between August 1995 and December 2017, with an average observation period of 60 years following initial exposure. In the years following data collection, the local government implemented a tap water supply program, which was providing nearly 100% of all tap water in the study area by the late 1990s. Accordingly, the residents were exposed to arsenic-contaminated drinking water at home ranging from 0 to 3842.61 ug/L for an average of 41 years at recruitment.

### Data Collection

Basic demographic characteristics, including age, sex, marital status, education level, occupation, cigarette smoking habit, alcohol drinking habit, coffee drinking habit, tea drinking habit, other chemical exposure and medical drug use, and duration of drinking well water at home were collected using a questionnaire. Urothelial tract cancer diagnosis data were obtained from the National Cancer Registration Database and were verified against insurance claims data because Taiwan implemented single payer national health insurance. International Classification of Diseases, Tenth Revision, Clinical Modification (ICD-10-CM) codes C67 and C64–66 and C68 were used to define bladder cancer and upper urothelial tract cancers, respectively. By the end of 2017, 85 incident cases of urothelial tract cancer had been identified. Underground water and urine samples were collected only at the time of recruitment and were stored at − 20 °C until assays were performed. The arsenic concentration in the water was determined immediately after sample collection. The initial exposure was defined when the participant’s birth or moving into the village. Therefore, the observational exposure period was calculated in average of 40 years but as the longest as 60 years among the participants. The well water and the participant’s urine were collected at recruitment during 1992–1995 and measured upon transporting to the laboratory then. When the tap water system was introduced, all participants stopped drinking well water during 1990’s. The measurements of well water and urine were performed one time upon recruitment, 1992–1995. The cumulative arsenic exposure was calculated based on the arsenic level determined from well water multiplied the length of a participant residing at the household before tap water system implementation.

Urinary arsenic speciation was performed through high-performance liquid chromatography to separate the arsenic species. This was followed by inductively coupled plasma mass spectrophotometry (NexION 350X, PerkinElmer, USA) to determine the concentrations of the separated arsenic species. The detection limits of the urinary iAs metabolites were 0.4, 1.4, 1.4, and 1.0 ug/L for Asi^+3^, Asi^+5^, MMA, and DMA, respectively. A spiking analysis yielded an average recovery rate of 95.18% to 100.03%. SRM 2670a, a standard reference material, was used for validation, and the values were calculated within the suggested range. According to our review of the literature, the iAs metabolism pattern was further revealed through the primary methylation index (PMI), calculated as MMA/(Asi^+3^ + Asi^+5^), and the, calculated as DMA/MMA [21].

### Statistical Analyses

Numerical variables with apparent skewness were presented as the median value (first and third quartiles), and logarithmic transformation before analysis of variance (ANOVA) and Scheffe’s multiple comparison procedure was used to compare the differences between the study groups. The Cox proportional hazards model was used to determine the strength of the association between the study variables and the occurrence of urothelial tract cancers during follow-up and for adjustment of the effects from other selected variables. To account for the collinearity among arsenic concentrations, duration of arsenic exposure, and cumulative arsenic exposure, we constructed various regression models for each arsenic exposure metric assessment. The iAs metabolism patterns were further categorized on the basis of the medians of the PMI and SMI into four groups: low PMI/low SMI, low PMI/high SMI, high PMI/low SMI, and high PMI/high SMI. To estimate the arsenic exposure doses, water arsenic concentrations were categorized according to a review of the literature (0–10, 10.01–50, 50.1–100, and > 100 ug/L). The cumulative arsenic exposure was categorized into tertiles (0–0.550, 0.551–2.548, > 2.548 ppm-years for T1, T2, and T3, respectively). The urinary iAs metabolites were categorized into tertiles (< 46.832, 46.832–95.342, and > 95.342 ug/L for T1, T2, and T3, respectively). Accordingly, the low methylation capacity group contained metabolites whose PMIs or SMIs were lower than their respective median values, and the high methylation capacity group contained those whose PMIs and SMIs were higher than their respective median values. Data were analyzed using SAS 9.4.

## Results

The incidence density of urothelial tract cancers was 78.21 × 10^−5^/year and was significantly higher in the older age group (HR: 1.92; 95% CI, 1.24–2.98) than it was in the younger age group (Table 1). Water arsenic concentrations and cumulative arsenic exposure were significantly higher in the bladder cancer and upper urothelial tract cancers groups than they were in the non-cancer control group. Total urinary iAs metabolites, urinary iAs, and DMA were higher in the bladder cancer group than they were in the non-cancer control group (Table 2).

**Table 1:** Descriptive statistics of arseniasis cohort in association with urothelial tract cancers (*ICD10* = C64–68)

	Person-years	n	Incidence density	Univariate analysis
			(10^–5^/year)	HR (95% CI)
Sociodemographic variables				
Overall (*n = *5811)	108,683.65	85	78.21	
Age at recruitment				
< 58	63,428.12	38	59.91	1.00
≥ 58	45,255.53	47	103.85	1.919 (1.237, 2.977)
Sex				
Female	56,006.77	42	74.99	1.00
Male	52,676.88	43	81.63	1.11 (0.718, 1.708)
Marry status				
Married	105,968.97	84	79.27	1.00
Single	1888.49	1	52.95	0.723 (0.101, 5.188)
Education				
Elementary and lower	100,234.16	80	79.81	1.00
Junior high and higher	8147.34	4	49.10	0.61 (0.222, 1.655)
Occupation				
Soldiers/government employees	1255.63	1	79.64	1.26 (0.546, 4.51)
Labor workers and business person	15,518.46	13	83.77	1.33 (0.549, 2.699)
Agriculture, and fisherman	46,068.25	42	91.17	1.45 (0.859, 2.336)
Housekeeping and others	39,675.00	25	63.01	1.00
Lifestyle variables				
Cigarette smoking				
No	66,706.98	45	67.46	1.00
Yes	41,976.68	40	95.29	1.45 (0.94, 2.243)
Alcohol drinking				
No	88,977.83	70	78.67	1.00
Yes	19,573.7	15	76.63	0.98 (0.551, 1.742)
Tea				
No	82,541.27	62	75.11	1.00
Yes	25,842.75	23	88.99	1.24 (0.766, 2.007)
Exercise				
No	84,732.74	62	73.17	1.00
Yes	23,614.93	23	97.40	1.304 (0.794, 2.143)

**Table 2:** Association of arsenic exposure metrics among study groups

	Non-cancer controls (*n = *5726)	Bladder cancer cases (ICD10 = 67) (*n = *49)	HR	Upper urothelial tract cancers (ICD10 = 64–66, 68) (*n = *36)	HR	ANOVA^a^
	Means $\pm$ SD	Means $\pm$ SD		Median $\pm$ SD		*p* value
Arsenic exposure						
Concentration in well water (ug/L)	133.28 $\pm$ 302.24	350.45 $\pm$ 696.36	1.00*	346.23 $\pm$ 643.20	1.00*	< .0001^bc^
Years of exposure	41.54 $\pm$ 16.13	44.33 $\pm$ 15.10	1.02	41.14 $\pm$ 14.76	1.00	0.585
Cumulative arsenic exposure (ppm-years)	4.44 $\pm$ 10.58	10.08 $\pm$ 17.35	1.02	13.11 $\pm$ 24.19	1.03*	< .0001^bc^
Urinary inorganic arsenic metabolites						
Total (ug/L)	99.10 $\pm$ 130.43	192.48 $\pm$ 244.32	1.00*	95.95 $\pm$ 76.45	1.00	0.078
Inorganic arsenic (ug/L)	9.32$\pm$ 13.11	27.94 $\pm$ 65.29	1.02*	13.16 $\pm$ 17.77	1.01	0.064
MMA (ug/L)	10.72 $\pm$ 18.64	17.77 $\pm$ 26.68	1.01	11.45 $\pm$ 13.68	1.00	0.508
DMA	79.07 $\pm$ 110.51	146.77 $\pm$ 174.49	1.00*	71.34 $\pm$ 56.83	1.00	0.098
Asi%	11.90 $\pm$ 9.74	12.58 $\pm$ 8.71	1.01	15.44 $\pm$ 14.34	1.02	0.598
MMA%	9.94 $\pm$ 6.66	9.09 $\pm$ 5.90	0.98	10.61 $\pm$ 7.76	1.01	0.577
DMA%	78.15 $\pm$ 11.60	78.33 $\pm$ 11.10	1.00	73.95 $\pm$ 14.50	0.98	0.200
PMI	1.95 $\pm$ 7.17	1.55 $\pm$ 2.86	0.98	4.50 $\pm$ 15.63	1.01	0.633
SMI	14.80 $\pm$ 62.39	19.41 $\pm$ 36.65	1.00	10.71 $\pm$ 6.57	0.99	0.506

*p < 0.05, performed by univariate Cox regression model

^a^Logarithmic transformation before ANOVA and Scheffe’s multiple comparison procedure

^b^Statistical significance when comparing bladder cancer cases and non-cancer controls

^c^Statistical significance when comparing upper urothelial tract cancer cases and non-cancer controls

^d^Statistical significance when comparing upper urothelial tract cancer cases and bladder cancer cases

After adjustment for age, sex, marital status, education level, occupation, cigarette smoking habit, alcohol drinking habit, and iAs metabolism patterns (PMI and SMI), the Cox regression analyses revealed a significant association between water iAs concentrations > 100 ug/L and the occurrence of bladder cancer at a HR of 4.88 (95% CI 1.35–17.61). In addition, a statistically significantly monotonic trend was observed between water iAs concentrations and upper urothelial tract cancers. A dose–response relationship was observed between cumulative arsenic exposure and bladder cancer but not between cumulative arsenic exposure and upper urothelial tract cancers. A significant monotonic trend was observed between total urinary iAs metabolite tertiles and bladder cancer (Table 3).

**Table 3:** Multiple Cox regression analyses for arsenic exposure metrics and urothelial tract cancers

	Bladder cancer (ICD10 = C67)						Upper urothelial tract cancers (ICD10 = C64–66, 68)					
	Model I		Model II		Model III		Model I		Model II		Model III	
	HR	(95% CI)	HR*	(95% CI)	HR*	(95% CI)	HR*	(95% CI)	HR*	(95% CI)	HR*	(95% CI)
Arsenic exposure												
Concentration in well water (ug/L)												
$\le$ 10.00	1*	–					1*	–				
10.01 ~ 50.00	1.86	(0.49, 7.04)					0.40	(0.10, 1.68)				
50.01 ~ 100.00	1.61	(0.27, 9.67)					2.08	(0.60, 7.26)				
> 100.00	4.88	(1.35, 17.61)					1.63	(0.51, 5.20)				
Cumulative arsenic exposure (ppm-years; in tertile)												
$\leq$ 0.555			1*	–					1	–		
0.556 ~ 2.548			5.06	(1.09, 23.49)					0.50	(0.13, 1.93)		
> 2.548			10.22	(2.30,45.47)					2.13	(0.80, 5.64)		
Urinary inorganic arsenic metabolites (ug/L)												
$\leq$ 46.832					1*	–					1	–
46.833– 95.342					2.64	(0.80, 8.75)					7.55	(1.65, 34.53)
> 95.342					3.79	(1.15, 12.47)					4.28	(0.83, 22.11)
Arsenic methylation capacity												
PMI (median low/high)												
High	1	–	1		1	–	1		1	–	1	–
Low	0.60	(0.26, 1.39)	0.61	(0.26, 1.43)	0.45	(0.19,1.09)	0.57 (0.23,1.43)		0.56	(0.22, 1.40)	0.48	(0.18, 1.24)
SMI(median low/high)												
High	1	–	1	–	1	–	1	–	1	–	1	–
Low	1.18	(0.46, 3.02)	1.14	(0.44, 2.92)	1.00	(0.39, 2.59)	0.75	(0.28,1.97)	0.75	(0.29, 1.98)	0.61	(0.23, 1.63)

*P < 0.05, test for monotonic trend performed by multiple Cox regression analysis adjusted by age, sex, marital status, education, occupation, cigarette smoking, and alcohol drinking

**Fig. 1:**
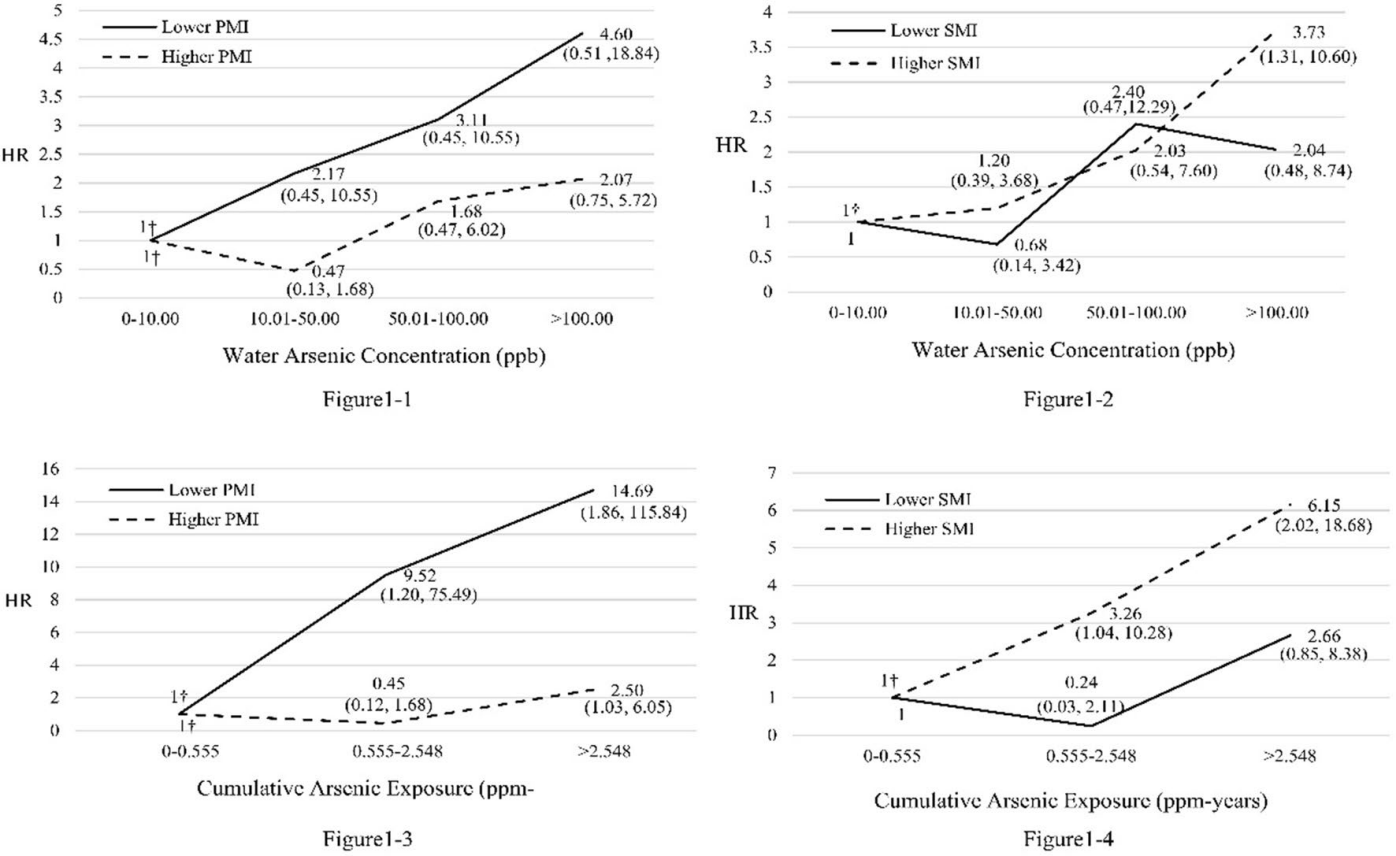
Association between arsenic exposure and incidence of urothelial tract cancers stratified by methylation capacity (adjusted for age, sex, marital status, education level, occupation, cigarette smoking habit, and alcohol drinking habit); 1–1. arsenic concentrations in water and incidence of urothelial tract cancers stratified by PMI (high vs. low); 1–2. arsenic concentrations in water and incidence of urothelial tract cancers stratified by SMI (high vs. low); 1–3. cumulative arsenic exposure and incidence of urothelial tract cancers stratified by PMI (high vs. low); 1–4. cumulative arsenic exposure and incidence of urothelial tract cancers stratified by SMI (high vs. low). †test for monotonic trend *p* < 0.05

Participants with exposure to water iAs concentrations of 0–10.00, 10.01–50.00, 50.01–100.00, and > 100.00 ug/L had HRs of 1.00, 2.17, 3.11, and 4.60 of developing urothelial tract cancers, respectively, in a dose–response relationship among participants with lower PMIs (*P* < 0.05 test for monotonic trend; Fig. 1). Participants with exposure to iAs concentrations in water of 0–10.00, 10.01–50.00, 50.01–100.00, and > 100.00 ug/L had HRs of 1.00, 1.20, 2.03, and 3.73 of developing urothelial tract cancers, respectively, in a dose–response relationship among participants with higher SMIs (*P* < 0.05 test for monotonic trend; Fig. 1). The tertile of cumulative arsenic exposure exhibited the same pattern of association between iAs concentrations and of developing urothelial tract cancers (Fig. 1).

## Discussion

This study demonstrated the occurrence of urothelial cancers, including those at upper urothelial tract and bladder sites, in an arseniasis community cohort after a 25-year cessation of drinking well water. The analyses revealed a dose–response relationship between low-to-moderate arsenic exposure and urothelial tract cancers among residents with lower PMIs or higher SMIs. However, the strength of the association between arsenic exposure and different sites of urothelial tract cancers, namely bladder and upper urothelial tract cancers, varied. The reasons for this varying carcinogenic potency remain unclear. The underlying mechanisms of arsenic carcinogenesis at organ sites, such as the lungs, skin, bladder, and upper urothelial tract, may differ with the dose in target cells, the molecular response to toxins, and local metabolism. According to the literature, these differences are primarily associated with concentrations of oxygen, arsenic species in the tissues, chemicals involving arsenic metabolism, and other endogenous cellular factors [20]. In addition, this study demonstrated that individuals with lower primary methylation or higher secondary methylation exhibited a dose–response relationship between low-to-moderate arsenic exposure, <100 ug/L, and urothelial tract cancers. This observation echoes those of advocates of the hazard of low-to-moderate arsenic exposure and indicates the key factors for assessing susceptibility. Accordingly, this finding provides a reference for future estimations of the risk of carcinogenesis due to low-to-moderate iAs exposure and a revision of the maximum allowance of arsenic in drinking water for susceptible populations.

Most epidemiological reports have focused on the association between arsenic exposure and bladder cancer, and only a few studies have discussed the effects of such exposure on upper urothelial tract cancers. In addition, a clear association between arsenic exposure, particularly low-to-moderate-level exposure, and higher upper urothelial tract cancers still requires validation [22–24]. Establishing an association between low arsenic exposure and a relatively lower incidence of cancer at organ sites is hindered by relative risks of nearly 1.0, insufficient statistical power, the necessity of managing confounding, exposure misclassification, and observation latency [25]. Therefore, although the IARC considers the evidence to date sufficient to support arsenic as increasing the risk of urinary bladder cancer, risks related to exposure in the 10–100 μg/L arsenic range remain unclear [8]. The patterns identified in this study were similar to those of a Chilean study, which demonstrated a considerable need for health care related to upper tract urothelial cancer and high mortality rates among residents who had been exposed to arsenic 25 years after the arsenic levels in drinking water had been controlled [26]. In addition, the present study contributes evidence of an association between exposure to low-to-moderate arsenic concentrations and the incidence of urothelial tract cancers in an exposure cohort. Our findings have supplemented the ecological literature on the effects of low-to-moderate arsenic concentrations, which includes a study demonstrating an association between low arsenic concentrations in drinking water (1.5–15.4 μg/L) and bladder cancer incidence in the United States [14] and a case–control study investigating the association between lifetime cumulative arsenic levels consumed through drinking water at a low-to-moderate arsenic exposure level and the incidence of bladder cancer [27]. This study has expanded on the understanding of this association by including an extended observation period and the participants’ methylation capacity to demonstrate the presence of a dose–response relationship between low-to-moderate iAs exposure and the risk of developing urothelial tract cancers.

Studies have demonstrated that a higher percentage of methylated arsenic in urine indicates a higher risk of skin and bladder cancer [17, 18]. Our previous cohort study demonstrated a dose–response relationship between arsenic exposure levels of 2–200 ug/L and lung cancer under conditions of lower methylation capacity. The present study demonstrated that the association between low-to-moderate arsenic exposure and urothelial tract cancers was noticeable among participants with lower PMIs or higher SMIs. The methylation capacity of exposed individuals was a key moderator of the risk of developing urothelial tract cancers. This observation supports the proposition that differing iAs methylation capacities is a main factor causing variability in arsenic carcinogenesis. Researchers have proposed that high methylation capacity is a protective mechanism against cancer development at skin, lung, and urothelial tract sites [23, 28, 29]. However, frequently high levels of methylation in cells are hazardous. In an animal model with urothelial carcinogenesis, arsenic was reported to be more hazardous in hosts with a methyl donor or glutathione depletion [30]. Accordingly, experiments in human cells have demonstrated that arsenic depletes cellular S-adenosylmethionine concentrations and causes DNA hypo-methylation. Low methylation capacity and high methylation demands may cause the accumulation of toxic arsenicals and in turn result in genotoxic events, such as the induction of oxidative stress, interference with signal transduction, and gene expression [31]. Furthermore, an animal model demonstrated that genome-wide DNA hypo-methylation caused by relentless iAs methylation may provide the basis for improving the understanding of human carcinogenesis [32]. These mechanisms explain why a higher risk of developing urothelial tract cancers was observed among individuals with lower PMIs or higher SMIs in this study; relatively lower MMA metabolites may be a key indicator in iAs carcinogenesis of urothelial tract cancers. The epidemiological data with a sufficient follow-up period in this study offer a valuable opportunity for verifying these mechanisms.

Previous studies have demonstrated that smoking is a risk factor for urothelial tract cancers including bladder cancers. In the present study, we have found a positive association between cigarette smoking and urothelial tract cancers but not statistically significant. Due to the long period of observation, the participants were beyond the population life expectancy. Smoking effect on urothelial tract cancers was confounded by many conditions such as competing causes of death in which the strength of association was underestimated. Moreover, we did not follow up the smoker’s status of their cessation behaviors of which may cause the insignificant result. We have reviewed previous Taiwanese studies; it is interesting to find that smoking effect was either treated as a control variable or a not significant factor to the urothelial tract cancers in Taiwanese populations [2, 3, 29]. Further studies are warranted in the arseniasis areas in Taiwan.

The strength of this study is its cohort design with a sufficient follow-up period and its low detection bias, with verification through health insurance claims. In addition, the length of observation (> 60 years after exposure inception), which continued after 25 years of drinking well water cessation, offered a valuable opportunity to investigate the association between cancers and early life exposure to arsenic. Although these findings are novel and noteworthy, the study contains limitations that necessitate caution in interpreting the results. First, the specimens used to identify urinary arsenic metabolites were collected upon the participants’ recruitment. This study has performed one time measurement on the urinary arsenic metabolites. Although studies have demonstrated the soundness of repeated measurements, verification using a population subset may improve the reliability of the dosimetry. Second, iAs metabolism profiles may vary across ethnicities; therefore, generalizations of these results to individuals in other countries should be cautiously made. Third, due to the length of follow-up and the investigation being conducted 25 years after cessation of exposure, various confounding factors may be present among participants with respect to lifestyle changes, medical treatment/seeking behaviors, and other interventions. Lastly, due to the constraint of initial questionnaire design, some factors, such as the ingestion of Chinese herbs, and occupational chemical exposures, were not collected. Therefore, further analyses of the possible exposures to aristolochic acid by Chinese herbs medication were unable to be addressed.

## Conclusion

This cohort study with long follow-up period offered a valuable opportunity to investigate the association between cancers and early life exposure to arsenic. The analyses revealed a dose–response relationship between low-to-moderate arsenic exposure and urothelial tract cancers among residents with lower PMIs or higher SMIs. This study also demonstrated that individuals with lower primary methylation or higher secondary methylation exhibited a dose–response relationship between low-to-moderate arsenic exposure, <100 ug/L, and urothelial tract cancers. These findings provide a reference for future estimations of the risk of carcinogenesis due to low-to-moderate iAs exposure and a revision of the maximum allowance of arsenic in drinking water for susceptible populations.

## Data Availability

The dataset used for this study is available through a request to the National Cancer Registration Database and were verified against national health insurance claims database.

## Abbreviations

HRs: Hazard ratios
CI: Confidence interval
iAs: Inorganic arsenic
IARC: International Agency for Research on Cancer
UUT-UC: Upper urinary tract urothelial carcinoma
ORs: Odds ratios
MMA: Methylarsonic acid
DMA: Dimethylarsinic acid
ICD-10-CM: International Classification of Diseases, Tenth Revision, Clinical Modification
Asi^+^^3^: Inorganic arsenite
Asi^+^^5^: Inorganic arsenate
PMI: Primary methylation index
SMI: Secondary methylation index
